# Neuroendocrine Cancer of the Breast: A Rare Entity

**DOI:** 10.3390/jcm9051452

**Published:** 2020-05-13

**Authors:** Azzurra Irelli, Maria Maddalena Sirufo, Luca Morelli, Carlo D’Ugo, Lia Ginaldi, Massimo De Martinis

**Affiliations:** 1 Medical Oncology Unit, Department of Oncology, AUSL 04, 64100 Teramo, Italy; azzurra.irelli@hotmail.it; 2 Department of Life, Health and Environmental Sciences, University of L’Aquila, 67100 L’Aquila, Italy; maddalena.sirufo@gmail.com (M.M.S.); lia.ginaldi@cc.univaq.it (L.G.); 3 Allergy and Clinical Immunology Unit, Center for the Diagnosis and Treatment of Osteoporosis, AUSL 04, 64100 Teramo, Italy; 4 Department of Pathology, S. Chiara Hospital, 38122 Trento, Italy; luca.morelli@apss.tn.it; 5 Radiotherapy Unit, Department of Oncology, AUSL 04, 64100 Teramo, Italy; carlo.dugo@aslteramo.it

**Keywords:** neuroendocrine breast cancer, breast carcinoma, mTOR, everolimus, NEBC

## Abstract

Neuroendocrine breast cancer (NEBC) is a rare histotype of breast carcinoma that presents, in most cases, positive hormone receptors and negative HER2. Indeed, the analysis of gene expression profiles revealed that NEBC belongs mainly to the luminal subtype. Cases of HER2-positive and triple-negative NEBC are rare. The cardinal treatment of early NEBC is surgery, similar to the treatment of invasive non-special histological type carcinoma. The use of radiotherapy follows the criteria applied in infiltrating breast cancer of non-special histotype. In the post-operative phase, therefore after the surgical treatment of mammary quadrantectomy, or mastectomy associated with homolateral sentinel lymph node removal ± axillary dissection, based on the histopathological characteristics of the tumor, the use of chemotherapy (anthracycline + taxane) and/or hormone therapy, whether or not associated with anti-HER2 therapy (trastuzumab) is the rule. Literature data report the use of cisplatin and etoposide, as in small cell lung cancers. Most of the information currently available derive from single case reports or a series of clinical cases; it follows the difficulty of formulating definite recommendations on the correct management of this histological type of breast cancer. This review describes available knowledge on this rare entity to improve the diagnostic and therapeutic strategies and offer insights to stimulate exploration of the many aspects still unknown.

## 1. Introduction

Breast cancer is the primary cause of death among Western women [1] and primary neuroendocrine breast cancers (NEBCs) are a rare subtype of breast cancer [2] that, according to the World Health Organization (WHO), represent 2–5% [3,4]. Neuroendocrine differentiation is observed in up to 20% of mammary carcinomas, so the real incidence of NEBCs is difficult to evaluate because immunohistochemical neuroendocrine markers are not usually used in the diagnosis of breast cancer [5].

The most recognized theory on the histogenesis of NEBC suggests its derivation from the divergent differentiation of a neoplastic stem cell in both epithelial and neuroendocrine cells [6,7]. Another theory hypothesizes a derivation from neural crest cells that migrate to the mammary glands or an origin from neuroendocrine cells present in breast tissue [8].

The observation that NEBCs often resemble breast tumors in their histological features supports the hypothesis that NEBCs derive from the differentiation of an epithelial progenitor cell. Breast cancer cells possess the ability to express neuroendocrine markers and benign neuroendocrine lesions of the breast have never been reported in the literature, unlike other organs, in particular the lung and gastrointestinal tract [7]. NEBC was first described in 1963 by Feyrter and Hartmann [9] and then in 1977, Cubilla and Woodruff reported eight cases of NEBC, providing a histopathological classification and a clinical and prognostic analysis of this subtype of breast cancer [10,11]. In 2003, the WHO defined NEBC as a separate subtype of breast cancer [6].

## 2. Diagnosis

NEBC is defined as a carcinoma that has morphological features similar to neuroendocrine neoplasms of other organs. Morphologically, it grows forming nests and trabeculae in the fibrovascular stroma; rosettes, palisade cells, and solid-papillary formations can also be identified [12].

**Figure 1:**
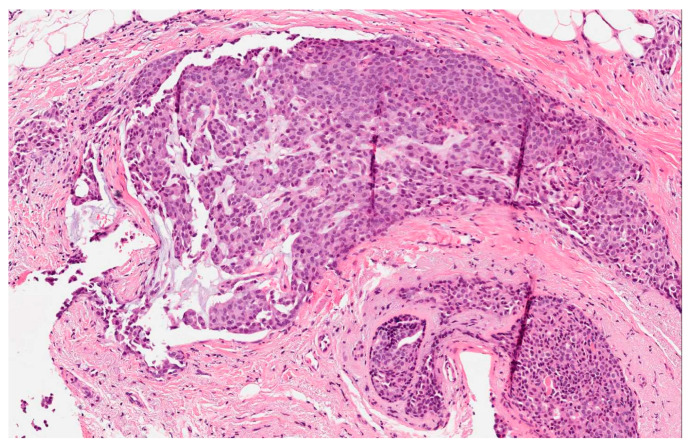
Hematoxylin-eosin (HE): neuroendocrine tumor of the breast. The cells appear polygonal-oval with eosinophilic cytoplasm and round nuclei. Focuses of mucin are present within the lesion (40×).

The cytological characteristics vary and fall mainly into four models: groups of large cells that constitute nests; groups of small and medium-sized cells that form trabeculae/ribbons and glandular structures; mixed growth patterns; groups of cells rich in extracellular and/or intracellular mucin. Cancer cells are predominantly polygonal-oval, sometimes small, with eosinophilic/eosinophilic-granular or clear cytoplasm (Figure 1). The nuclei of tumor cells are mostly round and oval with uniformly distributed or finely granulated chromatin (“salt and pepper”) [11].

Finding mucin is known to be tightly related to neuroendocrine differentiation and may be present at variable rates [11].

Neuroendocrine differentiation in mucinous carcinoma occurs and is associated with more favorable histological and immunohistochemical parameters. In these cases, patients are older and show a higher percentage of tumors with a lower nuclear degree, a lower incidence of axillary lymph node metastases, a higher progesterone receptor, and a lower expression of HER2 [12]. In NEBCs, a low incidence of microcalcifications has been reported [13]. Specific histological types may present neuroendocrine differentiation such as hypercellular neuroendocrine carcinoma and solid-papillary carcinoma [13].

### 2.1. Definition of Neuroendocrine Breast Cancer

According to WHO guidelines, the combination of morphology and immunohistochemistry allows the diagnosis of neuroendocrine carcinomas [14,15].

**Figure 2:**
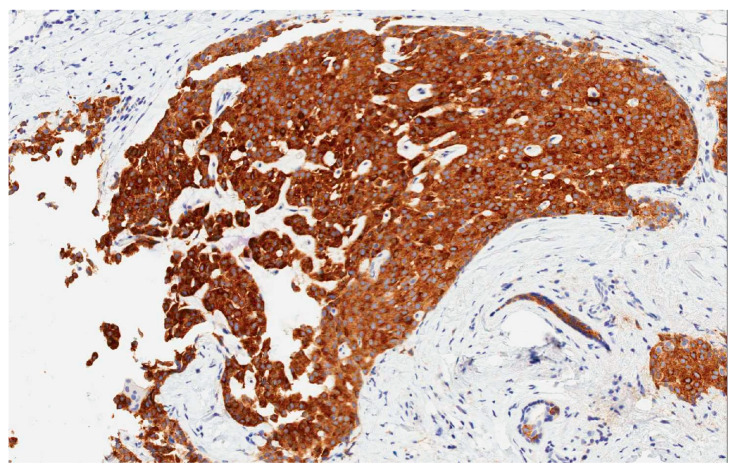
Immunohistochemistry (ICH): neuroendocrine tumor of the breast. Strong immunohistochemical expression of synaptophysin (40×).

Initially, the WHO identified three criteria to define NEBC:

1. The presence of over 50% of neoplastic cells expressing neuroendocrine markers of immunohistochemistry such as chromogranin A and synaptophysin (Figure 2). Neuron-specific enolase (NSE) and CD56 appear to result in lower sensitivity and specificity, mostly because they are normally present in breast tissue [2,5,6,11]. When the neuroendocrine characteristics are shown in less than 50% of cancer cells, the tumor should be identified as a breast cancer with neuroendocrine differentiation. The focal neuroendocrine differentiation within the carcinoma of the breast is common and has no prognostic significance.
2. Excluding primary extra-mammary tumors, especially of the lung and the gastroenteric tract.
3. The identification of a concomitant component in situ in the breast [6].

In 2012, the WHO established that the 50% threshold of neoplastic cells expressing immunohistochemical neuroendocrine markers was arbitrary; therefore, invasive carcinomas with neuroendocrine differentiation were included in the NEBC group regardless of the percentage of tumor cells expressing neuroendocrine markers of immunohistochemistry [5,13], after excluding other primary tumor sites.

The 2012 WHO classification of breast carcinomas classifies neuroendocrine tumors into three subtypes: well-differentiated neuroendocrine tumors; poorly differentiated neuroendocrine carcinomas or small cell carcinomas; and invasive breast carcinomas with less neuroendocrine features of 50% of cancer cells [2,11].

In 2019, there was a radical change in the WHO classification of neuroendocrine tumors/carcinomas to create a common way of classification across sites to reduce inconsistencies and contradictions among the various systems currently in use.

Neuroendocrine neoplasm is intended as a definition that comprises all tumor classes with predominant neuroendocrine differentiation including both well differentiated and scarcely differentiated types. Neuroendocrine tumor is an invasive tumor characterized by: low/intermediate grade, neuroendocrine morphology, and supported by the presence of neurosecretory granule and diffuse, uniform immunoreactivity for neuroendocrine markers. The main prognostic parameters used are tumor stage and histological grade, which include mitotic counts [16].

### 2.2. Characteristics

**Figure 3:**
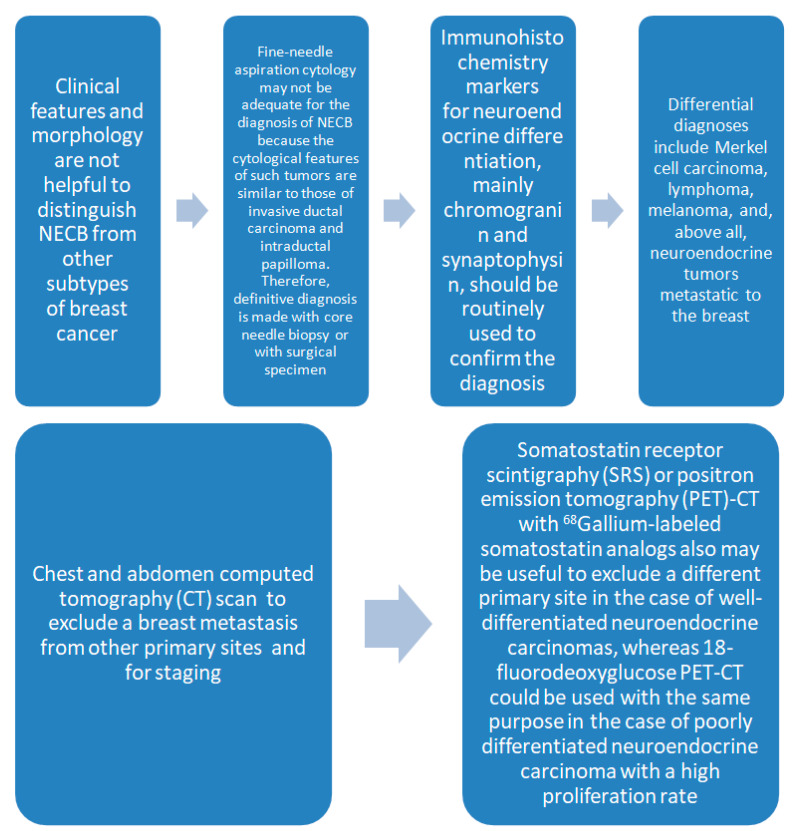
The diagnostic process in neuroendocrine breast cancer.

Neuroendocrine tumors exhibit nested or trabecular and low-grade growth, and express neuroendocrine markers in a widespread manner; they typically express the estrogen receptor, have a low ki67, and are HER2-negative. Neuroendocrine carcinomas, on the other hand, are poorly differentiated and are similar to small cell carcinoma and large cell carcinoma [17]. Cloyd et al. found a prognostic value in NEBC histology, which is related to an advanced stage at diagnosis and unfavorable prognosis for the second to fourth stages [14]. NEBC possess clinical features indistinguishable from other carcinomas. Indeed, NEBC is found as a palpable or non-palpable mammary nodule, identifiable by ultrasonography, mammography, or magnetic resonance imaging. It has no unique radiological features and the diagnosis is made on histology, which, in turn, is based on obtaining adequate tissues and suitable for histological analysis. Tissues that show histological aspects suggestive of neuroendocrine differentiation should therefore undergo immunohistochemical staining for neuroendocrine markers to endorse the diagnosis (Figure 3). NEBC must be distinguished from adenocarcinoma, lymphoma, sarcoma, Merkel cell carcinoma, malignant melanoma, and metastatic neuroendocrine breast tumors [6,7,13]. Brask et al. suggested tissue microarray analysis as a useful tool to identify NEBC [18]. The possibility of breast metastases from other primitive neuroendocrine cancers should be denied performing a CT scan. Somatostatin receptor scintigraphy (SRS) or positron emission tomography (PET)-CT with gallium-labeled somatostatin analogues 68 may also be indicated for differentiation from a diverse primitive site in the case of well-differentiated neuroendocrine carcinomas, whereas PET-CT with 18-fluorodeoxyglucose could be performed with similar intention in the event of scarcely differentiated neuroendocrine carcinoma with a high proliferation rate [19].

Exceptionally, these tumors, mainly occurring in post-menopausal women, may be associated with functional syndromes [2,13]. The mammary origin of neuroendocrine tumors can be advocated when a ductal component is found in situ and/or immunohistochemical stains demonstrate positivity for CK7 while the negative stains each for TTF-1, CDX2, PAX8/PAX6 exclude the lung, gastrointestinal, and gastropancreatic/gastroduodenal tract origin, respectively [7,20]. Estrogen receptor (ER) positivity alone is not enough to define the mammary nature of a neoplasm as it is neither universally expressed in normal breast cancer nor is it confined to mammary tumors. Where staining is not conclusive, GCDFP-15 and/or mammaglobin are adjunctive stains that can be used with good ability to distinguish for breast tissue. The expression of the androgen receptor was lower than that of non-special luminal invasive carcinomas, about 15–18% of NEBC [2]. Estrogen receptors are positive in most primary NEBCs, while HER2 is almost always negative [21].

### 2.3. Genetic Indications

Analysis of gene expression profiles revealed that NEBCs mostly belong to the luminal subtype [5,21,22]. A study showed that 88% of 112 patients with NEBC hold the luminal phenotype; in particular, luminal A (42%) and luminal B (58%) [23]. A similar distribution of luminal A (52%) and luminal B (48%) in NEBC was observed in another study [2]. A further study described primary breast cancer with neuroendocrine differentiation with a triple negative phenotype without baseline characteristics (negative immunoreactivity for cytokeratin 5/6, cytokeratin 14, and EGFR [20]), which indicated that the genes involved in the migration, invasion, and proliferation of neoplastic cells are under-regulated, giving NEBC a weak ability to metastasize and therefore the NEBC can be grouped with the mucinous carcinoma subtype [2,6]. On the other hand, it is known that, unlike non-special luminal invasive carcinomas, NEBCs have a significantly lower frequency of PIK3CA mutations. The presence of PIK3CA mutations confers clinical-pathological characteristics favorable to tumors such as small tumor size or low grade; and therefore, the reason for an unfavorable prognosis of NEBC is almost partially explained [2]. The literature reports PIK3CA mutations in 7–33% of cases [3]. NEBCs differ from the common forms of BC ER +/HER2− due to the low frequency of PIK3CA mutations (similar to mucinous carcinomas), but also due to the lack of TP53 mutations and the presence of FOXA1 mutations (similar to lobular carcinomas). Similar to the neuroendocrine tumors (NETs) of other sites, NEBCs also host mutations in chromatin remodeling genes including ARID1A and ATRX [24]. Some NEBCs also host FGRF and RAS mutations [25]. In NEBC have been found gene alterations in the cell cycle control pathway, suggesting the relevance of CDK4/6 inhibitors. NEBC patients are unlikely to benefit from immune checkpoint inhibitors, as all current biomarkers (PD-L1, TMB, and MSI) are uniformly negative in this rare histotype [3]. Xiang et al. identified clonal chromosomal aberrations in five out of seven NEBC cases, with four of them showing complex karyotypes. They also found that NEBC share some cytogenetic abnormalities such as trisomy 7 and 12 with other neuroendocrine tumors in the lung and gastrointestinal tract [26].

## 3. Prognostic Factors and Prognosis

Prognostic factors and predictive factors such as age of the patient, involvement of axillary lymph nodes, grading, hormonal receptors, Ki67, HER2, and intratumoral lymphocytes, stratify breast cancer patients, in general, into low, intermediate, and high risk, helping to estimate prognosis and predict response to treatments (Table 1). NEBC can metastasize to several sites (liver, bones, lung, pancreas, soft tissues, and brain or more rare sites like adrenal glands) even years after primary tumor treatment and therefore long-term follow-up is advisable [5]. Some studies [27,28] evidentiate that these cancers are clinically offensive, while other reports [29,30] describe a good prognosis. It should be emphasized that among these papers, several discrepancies should be underlined such as the numerical consistency of the reports, the follow-up time, type of immunohistochemical markers, and clone antibodies selected for diagnosis as well as the threshold value for positive staining within the tumor with these antibodies [11]. The prognostic relevance of these markers in these tumors is therefore debated. However, despite the luminal phenotype, most studies have reported an aggressive clinical course of NEBC [6,23] given the age, size, degree, and state of the estrogen receptor [2,21]. While other studies have reported a prognosis in NEBC similar to that for different invasive breast cancers and depends on staging, classification, mucin production, and apocrine tumor differentiation [6]. Small cell NEBC has a more favorable prognosis for disease status than small cell lung cancer. Recently, Roininen et al. assessed the immunohistochemical expression of well characterized prognostic factors of gastroenteropancreatic neuroendocrine tumors (GEP-NET) such as neuron specific enolase, thymidylate synthase, CD56, p27, menin, and somatostatin receptor type 2, and found a striking similarity in NEBC and GEP-NET. On the contrary, notable differences were found when compared with invasive ductal carcinomas [31].

### 3.1. Neuroendocrine Breast Cancer and the Somatostatin Receptors

Neuroendocrine tumors of lung, prostate, and gastrointestinal system as well as breast express somatostatin receptors (SSTRs) that have potent inhibitory effects on hormonal secretion and antiproliferative effects. Type 2A somatostatin receptors (SSTR2A) are associated with the strongest antiproliferative effects in vitro. A recent study demonstrated that the majority of primary NEBC express SSTR2A and SSTR5 with SSTR2 being the most consistently expressed [32]. So, as in other neuroendocrine tumor sites, SSTRs could be a potential therapeutic target. The relative high proportion of tumors negative for SSTRs, which may justify the therapeutic failure, suggest that it could be better to measure immunohistochemical SSTR status before commencing SSTR analogue treatment.

Based on the emerging evidence showing that receptor derived peptide fragments are involved in cancer development and progression, Del Rio Moreno et al. demonstrated that the truncated variant of somatostatin receptor subtype 5 (SST5TMD4) derived peptides could contribute to the strong oncogenic role of SST5TMD4 observed in multiple cancer pathologies and represent potential candidates to identify new diagnostic, prognostic, and therapeutic targets in oncology [33].

### 3.2. The PI3K Pathway

An important mutated pathway in tumors is the PI3K pathway [34], which has also been shown to have a relevant role in neuroendocrine tumors. Hyperactivation of this pathway may be highlighted immunohistochemically, staining the mammalian target of rapamycin (mTOR) protein (p-mTOR), a downstream protein of PI3K [35]. mTOR has a leading role in cell growth, nutrient signalling and inhibition of autophagy. These represent key processes in cancer development and progression [36]. Everolimus, a selective and potent inhibitor of mTOR, showed effectiveness across different types of neuroendocrine tumors [37]. Its effectiveness could also be hypothesized in NEBC with a role added to existing regimens. Everolimus, plus a steroidal aromatase inhibitor, is approved in recurrent or progressing hormone receptor positive advanced breast cancer. Therefore, studying the presence of p-mTOR in NEBC could help to select patients that could benefit from more complex therapeutic regimens.

## 4. Treatment

Neuroendocrine neoplasms of the breast are poorly defined and rare [38]. Poor data supporting the grading system as well as genetic knowledge in breast NETs, where available for existing classifications, hence the difficulty of being able to distinguish the various degrees of biological offensiveness and the diverse responses to pharmacological treatment. The recent WHO 2019 classification of neuroendocrine neoplasm had the purpose to help clinicians and pathologists to better manage their patients. Neuroendocrine tumors (well differentiated) (NETs) and neuroendocrine carcinomas (poorly differentiated) (NECs) share common ultrastructural, immunophenotypic, and histologic neuroendocrine features. These two groups of neoplasm, although having overlapping features in most organs, are not biologically closely related. Underpinning genetics, the relationship to non-neuroendocrine neoplasm, hereditary predisposition, and different risk factors distinguished NETs and NECs in the lung and digestive system while limited data are available about other systems. To date, no therapeutic significance is attributed to neuroendocrine differentiation in breast neoplasms. Currently, the same therapeutic approach applied in the treatment of different types of invasive breast cancer is used in neuroendocrine cases [17,39], and we do not have data from prospective clinical trials on their optimal management (Table A1) [16].

### 4.1. Surgery

**Figure 4:**
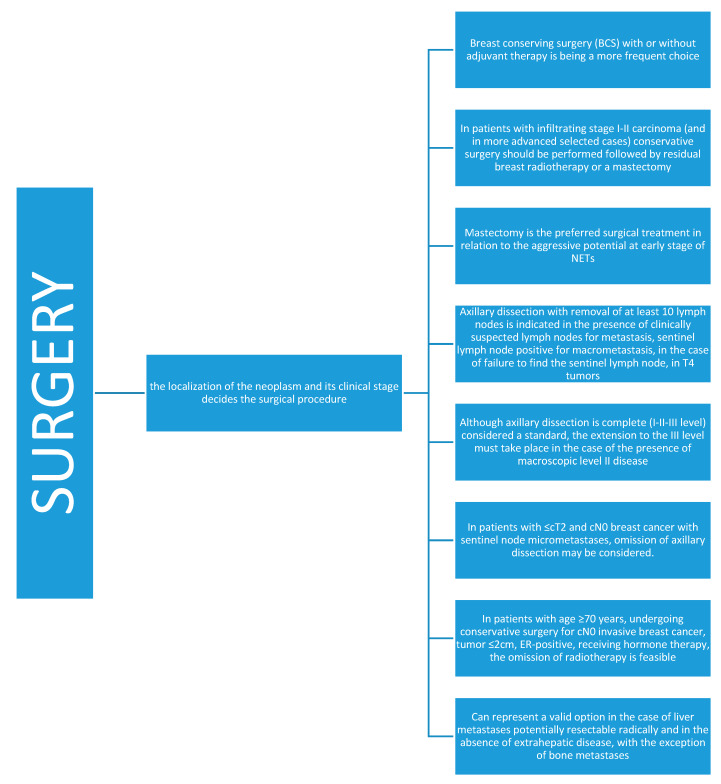
Surgery in neuroendocrine breast cancer.

Similar to most breast neoplasms, the recommended first line of treatment is surgery (Figure 4), but the rarity of this type of tumor has not allowed for the extent of the resection to be defined to date [7]. Available data in the literature, to date, are mainly based on the classifications prior to the WHO 2019 guideline, which could create a bit of confusion and lead to incorrect conclusions for the mixed nosological variety. Breast conserving surgery (BCS) with or without adjuvant therapy is a more frequent choice, but mastectomy is the preferred surgical treatment in relation to the aggressive potential at early stage of NETs [40,41]. We do not yet have enough information on which of the two surgical approaches is the best choice. Distinguishing between primary and metastatic NETs is essential to decide the better surgical approach, BCS or mastectomy and axillary node dissection [42]. A prerequisite for the best initial treatment approach in NEBC is a definite diagnosis and a complete stage [38]. Surgery is the cornerstone of the therapy of early NEBC and the localization of the neoplasm and its clinical stage decides the surgical procedure, similar to the primitive breast carcinomas of non-special histology. In patients with infiltrating stage I–II carcinoma (and in more advanced selected cases), conservative surgery should be performed, followed by residual breast radiotherapy or a mastectomy. Axillary dissection with the removal of at least 10 lymph nodes is indicated in the presence of clinically suspected lymph nodes for metastasis, sentinel lymph node positive for macrometastasis, in the case of failure to find the sentinel lymph node in T4 tumors and in inflammatory carcinoma. Although axillary dissection is complete (I–II–III level) and considered a standard, the extension to the III level must take place in the case of the presence of macroscopic level II disease. In patients with ≤cT2 and cN0 breast cancer with sentinel node micrometastases, omission of axillary dissection may be considered. In patients with age ≥70 years, undergoing conservative surgery for cN0 invasive breast cancer, tumor ≤2 cm, ER-positive, and receiving hormone therapy, the omission of radiotherapy is feasible.

### 4.2. Radiotherapy

**Figure 5:**
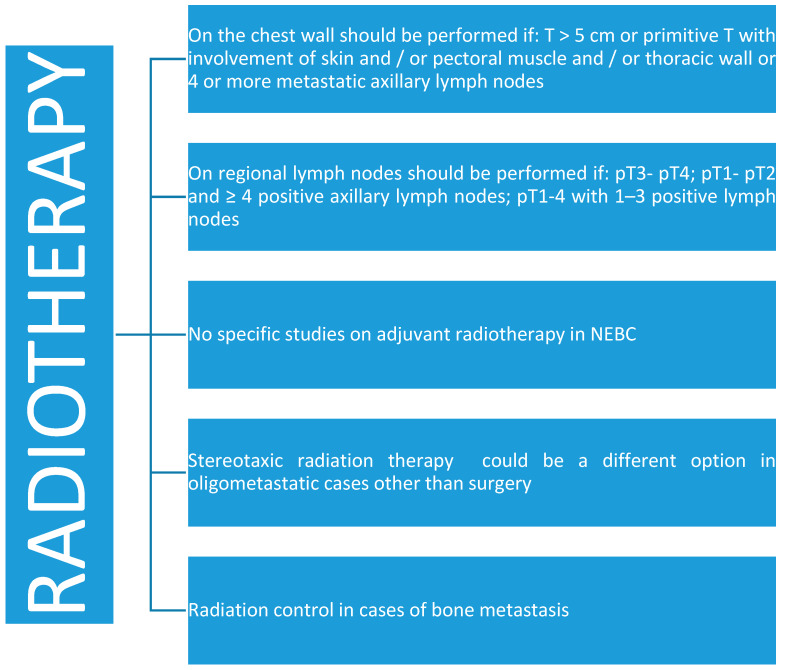
Radiotherapy in neuroendocrine breast cancer.

Radiotherapy (Figure 5) on the chest wall should be performed if: T > 5 cm or primitive T with involvement of skin and/or pectoral muscle and/or thoracic wall or four or more metastatic axillary lymph nodes. Radiotherapy on regional lymph nodes should be performed if: pT3-pT4; pT1-pT2, and ≥4 positive axillary lymph nodes; pT1–4 with 1–3 positive lymph nodes [17]. There are no specific studies on adjuvant radiotherapy in NEBC and it should be considered on the basis of recommendations given for different kinds of invasive breast cancer [4,5,6,22]. Hare et al. reported no benefit from radiotherapy in small cell carcinoma of the breast [42]. Wei et al. instead reported a benefit of adjuvant radiotherapy on survival, although not statistically significant [27].

### 4.3. Chemotherapy

It has been hypothesized that the breast NETs are chemoresistant, similar to pulmonary and gastrointestinal NETs [19]. Adjuvant systemic therapy must be decided in a personalizing view, taking into account the age of the patient, their comorbidities, the stage and the biological characteristics of the disease that identify the risk of relapse [4,5,6,22]. On the basis of immunohistochemistry, the subgroups of infiltrating breast cancer can be identified: luminal A, luminal B/HER2 negative, luminal B/HER2 positive, HER2 positive (no luminal), and triple-negative [17]. Patients diagnosed with NEBC with positive hormone receptors are candidates to receive adjuvant endocrine therapy. As observed in many cases of NETs [43], adjuvant endocrine therapy is supposed to increase survival, but evidence of efficacy needs clinical trials and is to date only anedoctal [44].

**Figure 6:**
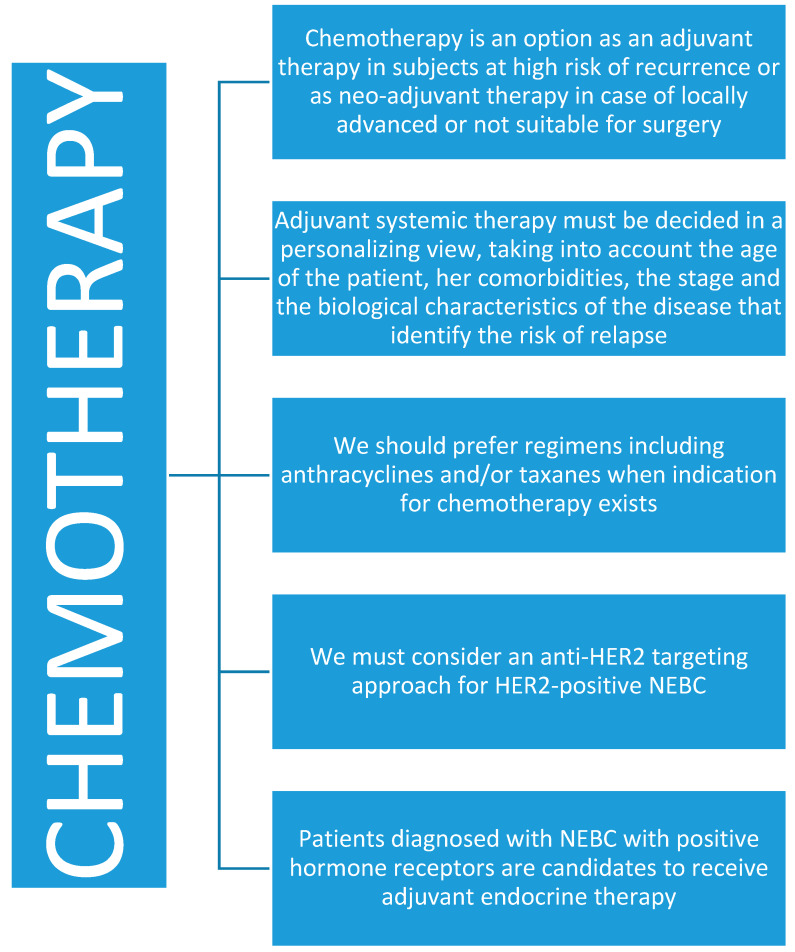
Chemotherapy in neuroendocrine breast cancer.

Chemotherapy (Figure 6) is an option as an adjuvant therapy in subjects at high risk of recurrence or as neo-adjuvant therapy in the case of locally advanced or not suitable for surgery NEBC. For the negative HER2 hormone-responsive disease, various analysis tests of gene profiles are available such as the Oncotype DX. The Oncotype Dx was built as a mathematical model that analyzes 21 genes, 16 of which inform about the proliferative state of the tumor and five control genes, and allows to divide patients operated on an early stage breast cancer into risk categories. By evaluating the differential expression of these genes, it is possible to associate each tumor with a continuous scale, a number, and a score which is associated with a prognosis. The score is called the Recurrence Score (RS) and predicts the risk of distant relapse within 10 years.

### 4.4. Patient Studies

Patients without lymph node metastases were enrolled in the TAILORx study; based on the RS value. They were divided into three groups: patients with RS ≤ 10, who undergo exclusive hormone therapy; patients with RS > 25, who undergo chemotherapy followed by hormone therapy; and patients with RS = 11–25, who are randomized to exclusive hormone therapy or to chemotherapy followed by hormone therapy. Data on patients with low RS have been published. At five years, the rate of DFS was 93.8%, the rate of patients free from relapse at a distance of 99.3% and the survival of 98% [38]. Low-risk women are generally less young with smaller, lower-grade tumors. The RxPONDER study, on the other hand, evaluates the use and therapeutic impact of the Oncotype Dx method for determining the risk of relapse in early-stage breast cancer with hormone receptor (HR)-positive, HER2 receptor negative and lymph node involvement up to three lymph nodes. We are currently waiting for the results [17,45]. In a limited collection of clinical cases, the Ki67 index was the basis for deciding the appropriate adjuvant chemotherapy, according to a therapeutic algorithm initially developed for neuroendocrine tumors of gastrointestinal origin. Subjects with Ki67 ≤ 15% received an anthracycline-based regimen and those with Ki67 > 15% received cisplatin/etoposide [5,30,46,47]. However, lacking reliable information on the role of platinum and etoposide for the adjuvant therapy of NEBC, it seems reasonable to use the same chemotherapeutic approach applied for ductal breast cancer, therefore, we should prefer regimens including anthracyclines and/or taxanes when an indication for chemotherapy exists. The significance of HER2 in establishing the prognosis in NEBC is not clear, but it can be assumed that it is similar to that suggested for different invasive breast cancers; therefore, we must consider ananti-HER2 targeting approach for HER2-positive NEBC. The use of somatostatin analogs has been reported in some studies [6]. Better knowledge of the molecular pathways involved in carcinogenesis could help identify new potential therapeutic targets [48,49,50].

### 4.5. The Therapeutic Decision in NEBC

Therefore, in general, in the decision-making process of the early NEBC therapeutic procedure, the histopathological parameters of the tumor such as T, N, grading, Ki67, hormonal receptors, HER2 status, and clinical parameters such as age, functional, and nutritional status and comorbidity must be considered.

When metastatic disease occurs, in particular, if methachronous, it would be advisable to carry out a biopsy of one of the metastatic sites in order to confirm the diagnosis and retype the disease from a biomolecular point of view, considering the possible heterogeneity between the primitive and metastatic site. Surgery can represent a valid option in the case of liver metastases potentially radically resectable and in the absence of extrahepatic disease, with the exception of bone metastases, which can be controlled by radiation therapy. Stereotaxic radiation therapy could be a different option in oligometastatic cases other than surgery. When, however, the disease is multi-metastatic, systemic treatment represents the forced choice. The chemotherapy regimens that can be used are those administered for different kinds of metastatic carcinoma and those administered for small cell lung cancer. For better indication refer to AIOM and NCCN guidelines [17,39].

### 4.6. Radionuclides and Other Treatments

For NEBC, which expresses somatostatin receptors, evidenced by the use of 68 Gallium PET-CT, receptor radionuclide therapy (PPRT) can be used [51]. A case of partial radiological response and complete biochemical response has been reported in the literature after treatment with 90Y-DOTATOC [52]. Activating mutations of VEGFR-2 and the high level of expression of VEGF-C have been found in NEBC and this provides the theoretical basis for investigating the use of antiangiogenic agents.

**Figure 7:**
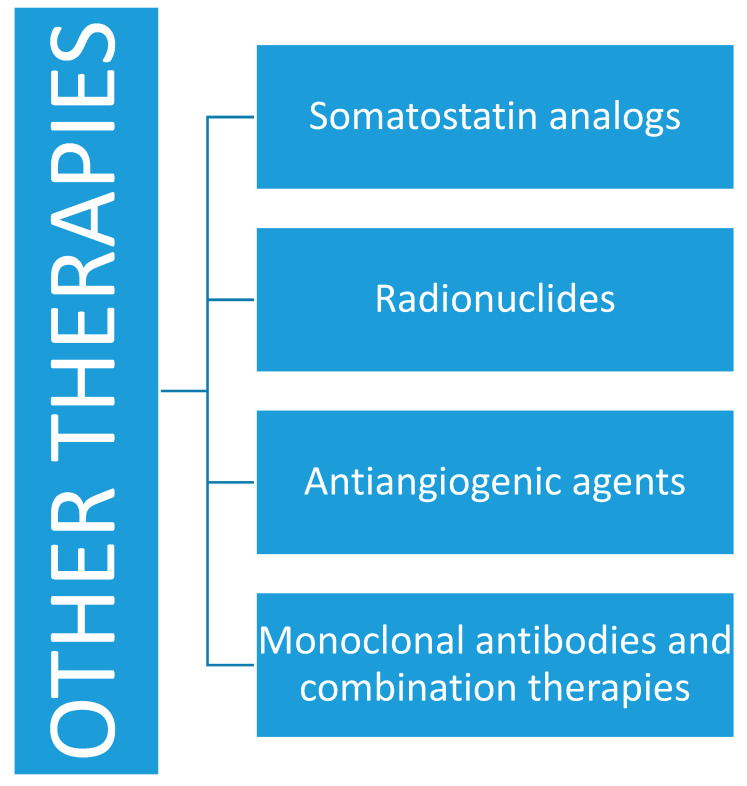
Other therapies in neuroendocrine breast cancer.

The most commonly evaluated monoclonal antibody was bevacizumab, which is a monoclonal antibody directed against VEGF, while the spectrum of TKI targeted to VEGFR was very broad, which includes numerous agents including sorafenib, sunitinib, and pazopanib. Furthermore, the use of new combinations between anti-VEGF therapies and systemic agents such as chemotherapy and hormone therapy are very interesting, given the different mechanisms of action of these agents [53,54] (Figure 7).

## 5. Conclusions

NEBC is a rare type of breast cancer. The recent classification of neuroendocrine neoplasms could be the basis for further improvement in the knowledge of these rare entities. Morphology was the primary basis of the classification and further genetic and pathology studies are indispensable for better characterizing these neoplasms and establish the clinical fallout from these aspects. Given the rarity of feedback, we do not have available literature data that direct us toward unambiguous therapeutic management and it is difficult to reach adequate conclusions on their best treatment strategy.

With the current small number of cases, it is unclear whether breast NETs should be managed following the guidelines of more common tumors. To date, there are no specific guidelines for their staging and treatment. It is recommended that they are treated similarly to other invasive breast carcinomas [17,39,55]. Currently, this remains an intriguing area of research and an increasing understanding of the biology of these rare tumors is necessary to develop the most appropriate therapeutic strategy. We hope this overview may be an opportunity to stimulate further investigation.

## Figures and Tables

**Table 1:**
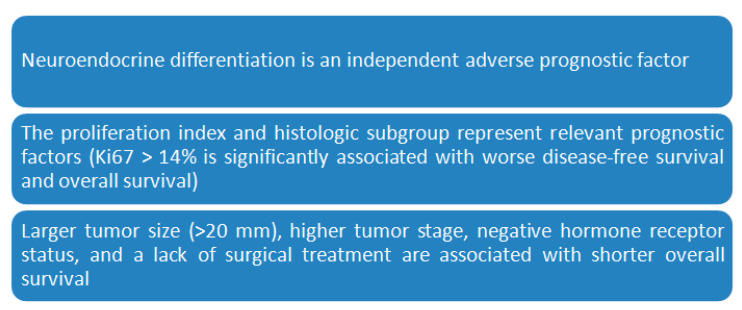
Prognostic factors in neuroendocrine breast cancer.

## Appendix A

**Table A1:** The table lists all studies published on PubMed since 2004. It is evident that these are mostly case reports. As illustrated in the text, there are limits relating to the uniformity of the diagnostic criteria up to the most recent WHO definitions. The most numerous studies are mostly retrospective in nature.

	STUDIES	TYPE OF STUDY	NUMBER OF CASES
1.	Cancers (Basel). 2020 Mar 20;12(3). pii: E733. doi: 10.3390/cancers12030733.	Clinicopathological study	5
2.	ClinNucl Med. 2020 May;45(5):e232-e235. doi: 10.1097/RLU.0000000000003005.	Case report	1
3.	Ann Ital Chir. 2020;91:23–26.	Clinicopathological study	11
4.	Rev Esp Med NuclImagen Mol. 2019 May - Jun;38(3):147–153. doi: 10.1016/j.remn.2018.11.007.	Imaging histopathological study	14
5.	Oncology. 2019;96(3):147–155. doi: 10.1159/000493348.	Immunohistochemical study	36
6.	Clin Breast Cancer. 2019 Apr;19(2):131–136. doi: 10.1016/j.clbc.2018.09.001.	Clinicopathological and genetic study	20
7.	Gan To Kagaku Ryoho. 2019 Dec;46(13):2222–2224.	Case report	1
8.	Pathol Int. 2019 Aug;69(8):502–504. doi: 10.1111/pin.12832.	Case report	1
9.	Gan To Kagaku Ryoho. 2018 Dec;45(13):2479–2481.	Case report	1
10.	Gan To Kagaku Ryoho. 2018 Dec;45(13):2432–2434.	Case report	1
11.	Gan To Kagaku Ryoho. 2018 Dec;45(13):1904–1906.	Case report	1
12.	Indian J EndocrinolMetab. 2018 May-Jun;22(3):308–315. doi: 10.4103/ijem.IJEM_446_17.	Clinicopathological study	2
13.	Ann DiagnPathol. 2018 Jun;34:122–130. doi: 10.1016/j.anndiagpath.2018.03.010	Clinicopathological study	36
14.	Gan To Kagaku Ryoho. 2018 Apr;45(4):682–684.	Case report	1
15.	Breast J. 2018 Jul;24(4):644–647. doi: 10.1111/tbj.12969	Case report	1
16.	Gan To Kagaku Ryoho. 2017 Nov;44(12):1592–1594	Case report	1
17.	BMJ Case Rep. 2018 Jan 4;2018. pii: bcr-2017-223280. doi: 10.1136/bcr-2017-223280.	Case report	1
18.	J Med Case Rep. 2017 Oct 19;11(1):290. doi: 10.1186/s13256-017-1467-0.	Case report	1
19.	Mod Pathol. 2018 Jan;31(1):68–82. doi: 10.1038/modpathol.2017.107.	Clinicopathological and genetic study	47
20.	Endokrynol Pol. 2017;68(5):597–602. doi: 10.5603/EP.a2017.0049.	Case report	4
21.	Int J Surg Case Rep. 2017;38:29–31. doi: 10.1016/j.ijscr.2017.07.002.	Case report	1
22.	Breast Dis. 2017;37(2):99–103. doi: 10.3233/BD-170283.	Case report	1
23.	Chin J Cancer. 2017 May 11;36(1):45. doi: 10.1186/s40880-017-0211-x.	Case series and literature search	7 + 119
24.	Pan Afr Med J. 2016 Nov 29;25:205. doi: 10.11604/pamj.2016.25.205.10366.	Case report	1
25.	Breast J. 2017 Sep;23(5):589–593. doi: 10.1111/tbj.12800.	Case report	1
26.	Breast Care (Basel). 2016 Dec;11(6):424–426. doi: 10.1159/000453572	Case report	1
27.	Gan To Kagaku Ryoho. 2016 Nov;43(12):2022–2025	Case report	1
28.	BMC Cancer. 2017 Jan 24;17(1):72. doi: 10.1186/s12885-017-3056-4.	Clinicopathological study	43
29.	J Pathol. 2017 Feb;241(3):405–419. doi: 10.1002/path.4837	Genetic study	18
30.	Ann Ital Chir. 2016 Nov 28;87. pii: S2239253X1602658X	Case report	5
31.	Breast Dis. 2016;36(4):161–164. doi: 10.3233/BD-160226.	Case report	1
32.	Conn Med. 2016 Oct;80(9):525–528.	Case report	1
33.	Pan Afr Med J. 2016 May 24;24:78. doi: 10.11604/pamj.2016.24.78.8546. eCollection 2016	Case report	1
34.	Mod Pathol. 2016 Aug;29(8):788–98. doi: 10.1038/modpathol.2016.69.	Clinicopathological study	22
35.	Indian J Cancer. 2015 Oct-Dec;52(4):636–7. doi: 10.4103/0019-509X.178432	Case report	1
36.	Int J SurgPathol. 2016 Aug;24(5):463–7. doi: 10.1177/1066896916632909.	Case report	1
37.	Indian J Cancer. 2015 Jan-Mar;52(1):85–6. doi: 10.4103/0019-509X.175563	Case report	1
38.	Gan To Kagaku Ryoho. 2015 Nov;42(12):1770-2	Case report	1
39.	J Breast Cancer. 2015 Dec;18(4):404–8. doi: 10.4048/jbc.2015.18.4.404	Case report	1
40.	Breast J. 2016 Jan-Feb;22(1):113–5. doi: 10.1111/tbj.12534. Epub 2015 Nov 25	Case report	1
41.	Breast Care (Basel). 2015 Aug;10(4):281–3. doi: 10.1159/000431070.	Case report	1
42.	J Med Assoc Thai. 2015 Aug;98(8):828–32.	Case report	1
43.	CeskPatol. 2015;51(3):128–31.	Case report	1
44.	Cancer Radiother. 2015 Aug;19(5):308–12. doi: 10.1016/j.canrad.2015.04.003.	Clinicopathological study	21
45.	Histopathology. 2016 Feb;68(3):422–32. doi: 10.1111/his.12766.	Clinicopathological study	128
46.	Springerplus. 2015 Mar 21;4:138. doi: 10.1186/s40064-015-0913-y. eCollection 2015.	Epidemiological study	199
47.	Breast J. 2015 May-Jun;21(3):303–7. doi: 10.1111/tbj.12403.	Case report	1
48.	Breast J. 2015 May-Jun;21(3):312–3. doi: 10.1111/tbj.12413. Epub 2015 Mar 23.	Case report	1
49.	Cytojournal. 2015 Jan 22;12:1. doi: 10.4103/1742-6413.149844.	Case report	1
50.	ApplImmunohistochemMolMorphol. 2015 Feb;23(2):97–103. doi: 10.1097/PDM.0b013e3182a40fd1.	Genetic study	9
51.	Bull Exp Biol Med. 2015 Jan;158(3):368–70. doi: 10.1007/s10517-015-2764-5.	Histopathological study	21
52.	Virchows Arch. 2015 Apr;466(4):479–81. doi: 10.1007/s00428-014-1704-5.	Case report	1
53.	Breast Cancer Res Treat. 2014 Dec;148(3):637–44. doi: 10.1007/s10549-014-3207-0.	Epidemiological study	248
54.	Histopathology. 2015 Apr;66(5):755–6. doi: 10.1111/his.12606	Histopathological study	59
55.	Int J Surg. 2014;12 Suppl 2:S8-S11. doi: 10.1016/j.ijsu.2014.08.392.	Case report	1
56.	Front Med. 2015 Mar;9(1):112–6. doi: 10.1007/s11684-014-0345-z.	Case report	1
57.	Hum Pathol. 2014 Sep;45(9):1951–6. doi: 10.1016/j.humpath.2014.06.002.	Cytogenetic study	7
58.	J Ultrasound Med. 2014 Aug;33(8):1511–8. doi: 10.7863/ultra.33.8.1511.	Imaging and Clinicopathological study	11
59.	AJR Am J Roentgenol. 2014 Aug;203(2):W221–30. doi: 10.2214/AJR.13.10749.	Imaging and Clinicopathological study	87
60.	Clin Imaging. 2014 Sep-Oct;38(5):734–8. doi: 10.1016/j.clinimag.2014.05.009.	Case report	1
61.	Int J Surg Case Rep. 2014;5(8):540–3. doi: 10.1016/j.ijscr.2014.05.006.	Case report	1
62.	Eur J GynaecolOncol. 2014;35(3):325–7.	Case report	1
63.	J BUON. 2014 Apr-Jun;19(2):419–29	Case report	1
64.	BMC Cancer. 2014 Mar 4;14:147. doi: 10.1186/1471-2407-14-147.	Epidemiological study	142
65.	Clin Breast Cancer. 2014 Oct;14(5):e99-e101. doi: 10.1016/j.clbc.2014.03.001	Case report	1
66.	Onco Targets Ther. 2014 May 6;7:663–6. doi: 10.2147/OTT.S60782.	Case report	1
67.	J Thorac Oncol. 2014 Jun;9(6):e40–2. doi: 10.1097/JTO.0000000000000103.	Case report	1
68.	ClinNucl Med. 2014 Apr;39(4):396–8. doi: 10.1097/RLU.0000000000000390.	Case report	1
69.	Gan To Kagaku Ryoho. 2013 Nov;40(12):2363–5	Case report	1
70.	J Clin DiagnRes. 2013 Nov;7(11):2585–6. doi: 10.7860/JCDR/2013/7051.3621	Case report	1
71.	Int J Surg. 2013;11 Suppl 1:S79–83. doi: 10.1016/S1743-9191(13)60023-0.	Epidemiological study	96
72.	J Breast Health. 2014 Oct 1;10(4):242–244. doi: 10.5152/tjbh.2014.1768	Case report	1
73.	J Breast Health. 2014 Apr 1;10(2):125–128. doi: 10.5152/tjbh.2014.1557	Case report	3
74.	J Breast Health. 2014 Apr 1;10(2):119–121. doi: 10.5152/tjbh.2014.1725	Case report	1
75.	Breast. 2014 Apr;23(2):120–7. doi: 10.1016/j.breast.2013.11.005.	Literature search	123
76.	APMIS. 2014 Jul;122(7):585–92. doi: 10.1111/apm.12197.	Epidemiological study	13
77.	APMIS. 2014 Jul;122(7):593–8. doi: 10.1111/apm.12198	Immunohistochemical study	18
78.	EndocrPathol. 2014 Sep;25(3):219–28. doi: 10.1007/s12022-013-9277-4.	Immunohistochemical and gene expression study	28
79.	Histopathology. 2014 Mar;64(4):597–600. doi: 10.1111/his.12276.	Case report	1
80.	J Coll Physicians Surg Pak. 2013 Nov;23(10):820–2. doi: 11.2013/JCPSP.820822.	Case report	1
81.	Histopathology. 2014 Apr;64(5):647–59. doi: 10.1111/his.12306.	Clinicopathological study	59
82.	Breast Dis. 2013 Jan 1;34(2):87–93. doi: 10.3233/BD-130356.	Clinicopathological study	15
83.	Breast Dis. 2014;34(3):95–9. doi: 10.3233/BD-130357.	Clinicopathological study	4
84.	World J Surg Oncol. 2013 Jun 5;11:128. doi: 10.1186/1477-7819-11-128.	Case report	1
85.	Breast J. 2013 Jul-Aug;19(4):382–7. doi: 10.1111/tbj.12121.	Clinicopathological study	22
86.	Korean J Radiol. 2013 May-Jun;14(3):395–9. doi: 10.3348/kjr.2013.14.3.395.	Case report	1
87.	PLoS One. 2013 Apr 22;8(4):e62487. doi: 10.1371/journal.pone.0062487.	Epidemiological study	34
88.	Pathology. 2013 Jun;45(4):422–4. doi: 10.1097/PAT.0b013e328360dfd1.	Case report	1
89.	J Coll Physicians Surg Pak. 2013 Apr;23(4):282–4. doi: 04.2013/JCPSP.282284.	Case report	1
90.	Breast J. 2013 Mar-Apr;19(2):204–6. doi: 10.1111/tbj.12081	Case report	1
91.	ZhonghuaZhong Liu ZaZhi. 2012 Dec;34(12):917–22. doi: 10.3760/cma.j.issn.0253-3766.2012.12.008.	Imaging and clinicopathological study	16
92.	Pan Afr Med J. 2012;13:40	Case report	1
93.	Clujul Med. 2013;86(2):156–9.	Case report	1
94.	Pan Afr Med J. 2013 Nov 11;16:92. doi: 10.11604/pamj.2013.16.92.2531.	Case report	1
95.	Neoplasma. 2013;60(2):215–22.	Clinicopathological study	107
96.	Anticancer Res. 2012 Dec;32(12):5227–32.	Immunohistochemical study	31
97.	CollAntropol. 2012 Sep;36(3):1053–5.	Case report	1
98.	Anticancer Res. 2012 Nov;32(11):5079–82.	Case report	1
99.	Am Surg. 2012 Nov;78(11):E457–8.	Case report	1
100.	Breast Care (Basel). 2012 Oct;7(5):408–10. doi: 10.1159/000343612.	Case report	1
101.	BMJ Case Rep. 2012 Aug 13;2012. pii: bcr1220115343. doi: 10.1136/bcr.12.2011.5343.	Case report	1
102.	Clin Imaging. 2012 Sep-Oct;36(5):599–601. doi: 10.1016/j.clinimag.2011.10.004.	Case report	1
103.	J CutanPathol. 2012 Nov;39(11):1042–6. doi: 10.1111/j.1600-0560.2012.01970.x.	Case report	1
104.	Breast Cancer. 2015 Jul;22(4):432-6. doi: 10.1007/s12282-012-0382-x.	Case report	1
105.	Ann Ital Chir. 2013 Jan-Feb;84(1):81–5.	Case report	1
106.	Chin J Cancer. 2012 Nov;31(11):549–56. doi: 10.5732/cjc.011.10370	Imaging and clinicopathological study	13
107.	J ClinPathol. 2012 Aug;65(8):699–703. doi: 10.1136/jclinpath-2012-200765.	Immunohistochemical study	3
108.	Pathol Int. 2012 May;62(5):331–4. doi: 10.1111/j.1440-1827.2012.02786.x	Case report	1
109.	Clin Breast Cancer. 2012 Aug;12(4):300–3. doi: 10.1016/j.clbc.2012.03.001	Case report	4
110.	Pathology. 2012 Apr;44(3):273–5. doi: 10.1097/PAT.0b013e3283513feb.	Case report	1
111.	Breast. 2012 Oct;21(5):652–6. doi: 10.1016/j.breast.2012.01.016.	Clinicopathological study	24
112.	J Korean Surg Soc. 2012 Feb;82(2):116–9. doi: 10.4174/jkss.2012.82.2.116.	Case report	1
113.	Zhonghua Bing Li XueZaZhi. 2011 Sep;40(9):604–9	Histopathological study	22
114.	Surg Today. 2011 Nov;41(11):1575–8. doi: 10.1007/s00595-010-4516-5.	Case report	1
115.	Neuro Endocrinol Lett. 2011;32(4):405–7.	Case report	1
116.	WMJ. 2011 Jun;110(3):140–3	Case report	1
117.	Breast Cancer. 2014 Jul;21(4):508–13. doi: 10.1007/s12282-011-0278-1	Case report	1
118.	Clin Breast Cancer. 2011 Oct;11(5):342–5. doi: 10.1016/j.clbc.2011.02.006	Case report	1
119.	J Coll Physicians Surg Pak. 2011 Jun;21(6):371–3. doi: 07.2011/JCPSP.371373.	Case report	1
120.	DiagnCytopathol. 2011 Jul;39(7):527–30. doi: 10.1002/dc.21494	Case report	1
121.	Histopathology. 2011 Jul;59(1):106–15. doi: 10.1111/j.1365-2559.2011.03880.x.	Histopathological study	74
122.	Ann Ital Chir. 2011 Jan-Feb;82(1):61–4.	Case report	1
123.	Surg Today. 2011 Jun;41(6):829–31. doi: 10.1007/s00595-010-4351-8.	Case report	1
124.	Int J ImmunopatholPharmacol. 2011 Jan-Mar;24(1):251–6.	Epidemiological study	23
125.	Hum Pathol. 2011 Aug;42(8):1169–77. doi: 10.1016/j.humpath.2010.11.014.	Clinicopathological study	74
126.	J ClinPathol. 2011 Jun;64(6):549–51. doi: 10.1136/jcp.2011.089219	Case report	1
127.	J Med Dent Sci. 2010 Jun;57(2):155–63.	Histopathological study	34
128.	Breast J. 2010 Nov-Dec;16(6):647–51. doi: 10.1111/j.1524-4741.2010.00974.x.	Case report	1
129.	Breast Cancer Res Treat. 2011 Feb;126(1):241–5. doi: 10.1007/s10549-010-1229-9.	Clinicopathological and histopathological study	8
130.	Ann Chir Plast Esthet. 2012 Dec;57(6):630–3. doi: 10.1016/j.anplas.2010.09.009.	Case report	1
131.	Breast Cancer Res Treat. 2010 Nov;124(2):413–7. doi: 10.1007/s10549-010-1178-3.	Histopathological study	9
132.	Surg Today. 2010 Sep;40(9):831–5. doi: 10.1007/s00595-009-4179-2.	Histopathological study	13
133.	Int J ClinExpPathol. 2010 Jun 30;3(6):629–33.	Case report	1
134.	J Med Case Rep. 2010 Jun 30;4:201. doi: 10.1186/1752-1947-4-201.	Case report	1
135.	Cancer. 2010 Oct 1;116(19):4463–73. doi: 10.1002/cncr.25352.	Case-controlled study	74
136.	Tunis Med. 2010 Apr;88(4):290–2	Case report	1
137.	Cytopathology. 2011 Feb;22(1):43–9. doi: 10.1111/j.1365-2303.2010.00742.x.	Cytopathological study	32
138.	Can J Surg. 2009 Dec;52(6):E289–90	Case report	1
139.	Breast Care (Basel). 2009 Nov;4(5):324–327. doi: 10.1159/000236226.	Case report	1
140.	Indian J Cancer. 2009 Oct-Dec;46(4):352–4. doi: 10.4103/0019-509X.55566	Case report	1
141.	Cancer Radiother. 2009 Dec;13(8):775–7. doi: 10.1016/j.canrad.2009.06.021	Case report	1
142.	Breast Cancer. 2012 Oct;19(4):360–4. doi: 10.1007/s12282-009-0160-6	Case report	1
143.	Mod Pathol. 2009 Nov;22(11):1401–14. doi: 10.1038/modpathol.2009.112	Genetic study	6
144.	Chir Ital. 2009 Mar-Apr;61(2):265–7	Case report	1
145.	ClinNucl Med. 2009 Feb;34(2):74–5. doi: 10.1097/RLU.0b013e318192c343	Case report	1
146.	Med MolMorphol. 2009 Mar;42(1):58–61. doi: 10.1007/s00795-008-0432-9.	Case report	1
147.	Breast J. 2009 Mar-Apr;15(2):205–6. doi: 10.1111/j.1524-4741.2009.00700.x	Case report	1
148.	Int J Surg. 2008;6 Suppl 1:S113–5. doi: 10.1016/j.ijsu.2008.12.007.	Observational study	35
149.	Indian J PatholMicrobiol. 2009 Jan-Mar;52(1):71–3.	Case report	1
150.	J Korean Med Sci. 2008 Dec;23(6):1118–20. doi: 10.3346/jkms.2008.23.6.1118.	Case report	1
151.	Indian J Med PaediatrOncol. 2009 Jan;30(1):31–4. doi: 10.4103/0971-5851.56334.	Case report	1
152.	Malays J Pathol. 2008 Jun;30(1):57–61.	Case report	1
153.	Oncol Rep. 2008 Dec;20(6):1369–74.	Clinicopathological study	12
154.	Anal Quant CytolHistol. 2008 Oct;30(5):306–8.	Case report	1
155.	Pathologica. 2008 Jun;100(3):176–80	Case report	2
156.	J Ultrasound Med. 2008 Sep;27(9):1401–5.	Case report	1
157.	Surg Today. 2008;38(8):734–8. doi: 10.1007/s00595-007-3716-0	Case report	1
158.	Histopathology. 2008 Sep;53(3):288–98. doi: 10.1111/j.1365-2559.2008.03093.x	Clinicopathological study	20
159.	Pathol Res Pract. 2008;204(11):851–5. doi: 10.1016/j.prp.2008.04.006.	Case report	1
160.	G Chir. 2008 May;29(5):203–6	Case series	10
161.	Cir Esp. 2008 May;83(5):273–4	Case report	1
162.	Clin Breast Cancer. 2007 Dec;7(11):892–4. doi: 10.3816/CBC.2007.n.056.	Case report	1
163.	Radiat Med. 2008 Jan;26(1):28–32. doi: 10.1007/s11604-007-0182-y.	Case report	1
164.	DiagnCytopathol. 2008 Jan;36(1):54–7.	Case report	1
165.	Am J Clin Dermatol. 2007;8(6):379–83.	Case report	1
166.	Tumori. 2007 Sep-Oct;93(5):496–8.	Case report	1
167.	Breast Cancer. 2007;14(4):414–9.	Case report	1
168.	Breast. 2008 Feb;17(1):111–4.	Case report	1
169.	Breast Cancer. 2007;14(2):250–3.	Case report	1
170.	Eur J GynaecolOncol. 2007;28(2):142–4.	Case report	1
171.	Indian J PatholMicrobiol. 2007 Jan;50(1):65–8.	Case report	1
172.	Int J Cancer. 2007 Jul 1;121(1):127–35.	Epidemiological study	76
173.	J ClinPathol. 2006 Aug;59(8):888	Case report	2
174.	Am Surg. 2006 Feb;72(2):185–7.	Case report	1
175.	Breast J. 2005 Nov-Dec;11(6):487	Case report	1
176.	J ClinPathol. 2005 Jul;58(7):775–8.	Case report	3
177.	J Exp Clin Cancer Res. 2004 Dec;23(4):691–6.	Case report	1
178.	Breast. 2004 Dec;13(6):527–9.	Case report	1
179.	Breast. 2004 Aug;13(4):329–33.	Clinicopathological study	9
180.	Eur J ObstetGynecolReprod Biol. 2004 Aug 10;115(2):231–3.	Case report	1
181.	J Nippon Med Sch. 2004 Jun;71(3):203–8.	Case report	1
182.	Ann Pathol. 2004 Jun;24(3):278–83	Case report	1
183.	Chin Med J (Engl). 2004 Oct;117(10):1536–40	Clinicopathological study	81
